# Gender and racial bias unveiled: clinical artificial intelligence (AI) and machine learning (ML) algorithms are fanning the flames of inequity

**DOI:** 10.1093/oodh/oqaf027

**Published:** 2025-10-09

**Authors:** Ahmed Umar Otokiti, Huan-ju Shih, Karmen S Williams

**Affiliations:** Digital Health 360 Degrees at Digital Health Solutions, LLC, 455 Tarrytown road, #1181, White Plains, NY, USA; Health Informatics, College of Public Health, George Mason University, 4400 University Dr, Fairfax, VA 22030, USA; Graduate School of Public Health and Health Policy, City University of New York, 55 W 125th St, New York, NY 10027, USA

**Keywords:** artificial intelligence, machine learning, model updating, data transparency, health equity, fairness and biases

## Abstract

This study aims to advocate for the continued evaluation of published clinical artificial intelligence (AI) and Machine Learning (ML) studies, including the reporting of demographic information, specifically gender, racial composition, and geographic location. Models are often trained on data lacking representation across basic demographics, potentially leading to biased outputs and exacerbating health disparities. Previous research in drug and device development has demonstrated the dangers of underrepresenting women and minority populations. This study aimed to assess the extent to which published clinical AI/ML studies report demographic information, specifically gender and racial composition, in their training datasets. A systematic review was conducted in accordance with PRISMA guidelines. The databases that were used in the study include Ovid MEDLINE, Embase, PsycINFO, Scopus, Web of Science and the Cochrane Library, for clinical AI/ML studies with direct implications for patient care. Inclusion criteria required models to be clinically actionable and not pre-clinical or administrative in scope. Two independent reviewers screened and extracted data using Covidence software, with conflicts resolved by a third reviewer. Out of 390 studies included, 84% of global models did not report the racial composition of their training data, while 31% lacked gender data. US-based models performed slightly better, with 56% reporting race and 77% reporting gender. Only 16% of all models utilized publicly available, non-proprietary datasets. The low frequency of demographic disclosure and limited use of open data raise serious concerns about the transparency, generalizability and fairness of clinical AI/ML models. Standardized reporting of gender and racial composition in training data is urgently needed to ensure ethical and equitable deployment of these technologies.

## INTRODUCTION

Biomedical drug and medical device development lacks gender, racial and geographic inclusivity [1, 2]. There are adverse consequences of inadequate inclusion of women and minority populations in clinical trials leading up to drug discovery [3, 4]. There is a surge in healthcare artificial intelligence (AI)/machine learning (ML) models with broad patient and population care applications. However, despite their great potential, AI/ML models are susceptible to cognitive, systemic and technical biases [5, 6]. There is a well-known systemic bias in the geographical distribution of patient cohorts used to train clinical models in the USA, with 70–90% from only three states, New York, California and Massachusetts [6]. The lack of geographical, gender and racial inclusivity in training data results in output bias and impaired model generalizability for safe deployment [5, 7]. We conducted a systematic review to examine the degree to which published clinical AI/ML models declare the gender and racial distribution of their training data.

## MATERIALS AND METHODS

**Figure 1:**
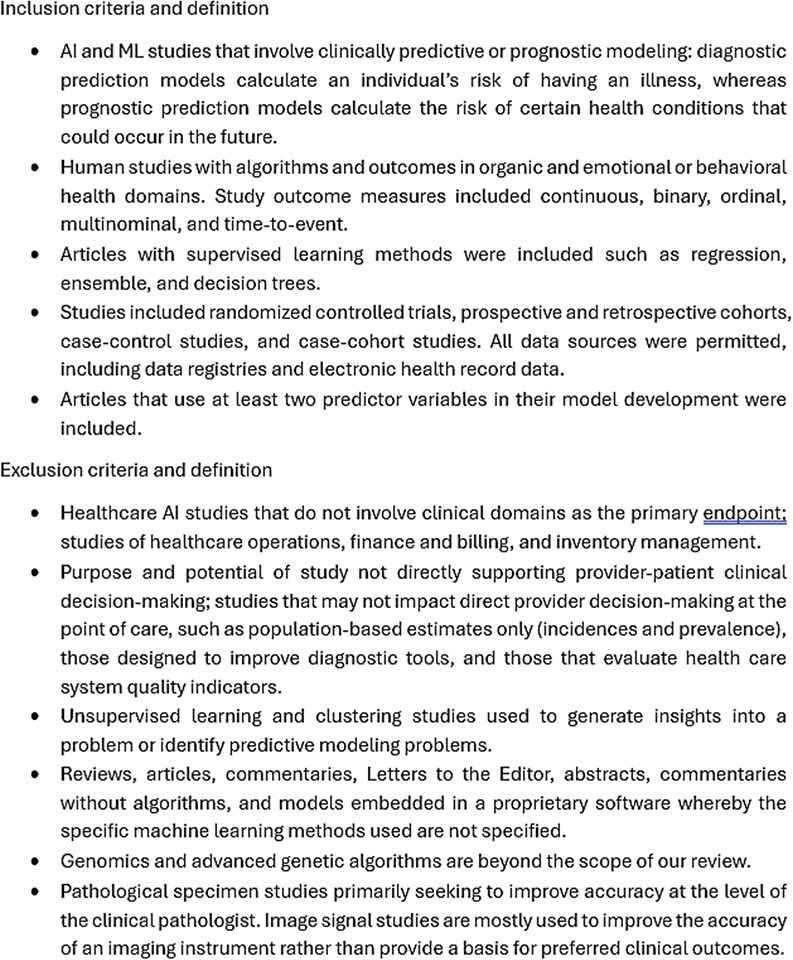
Inclusion and exclusion criteria.

**Figure 2:**
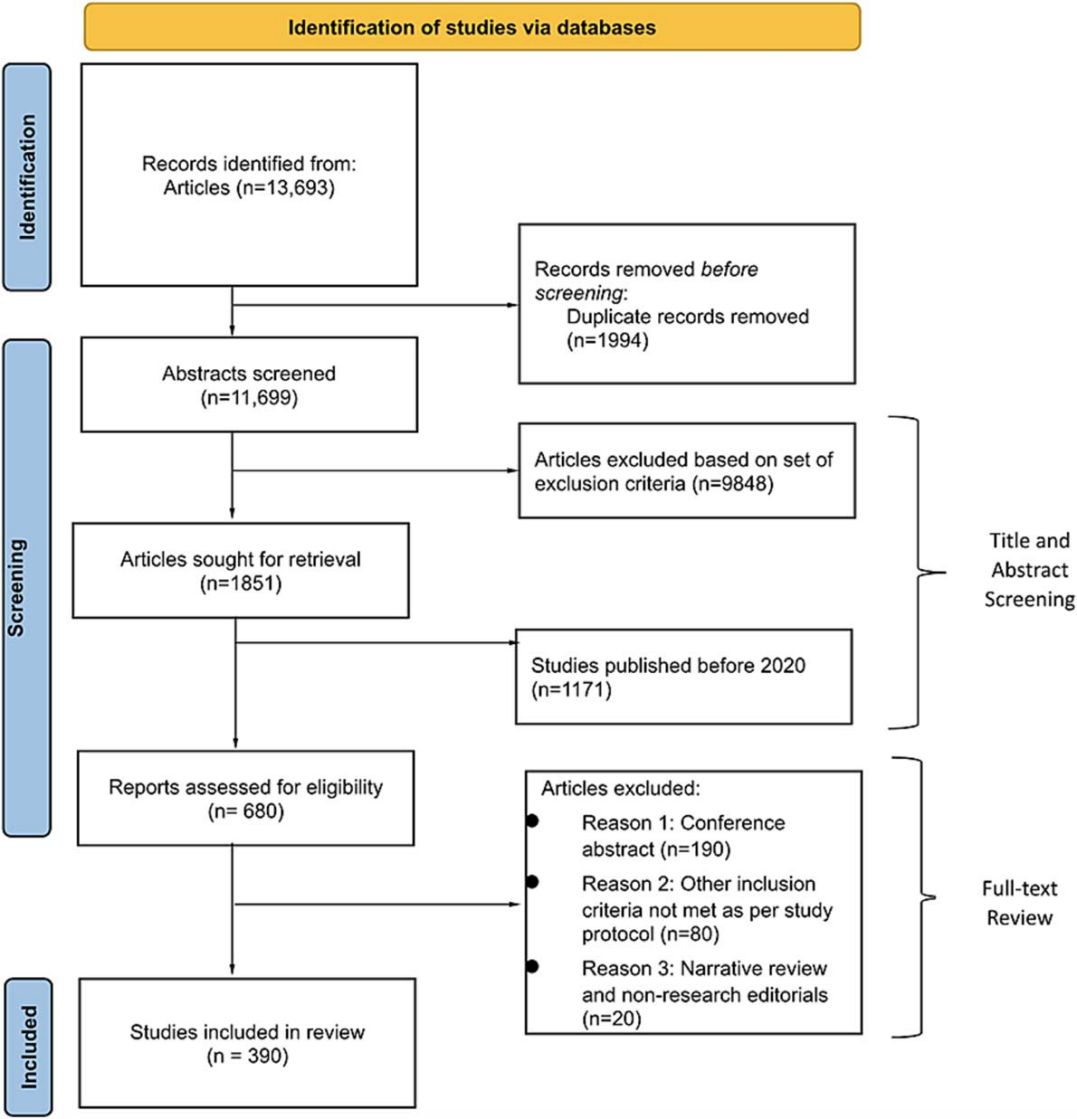
PRISMA flow chart.

Adhering to PRISMA guidelines, this study analyzed research papers on clinical AI/ML models that directly impact clinician decision-making at the level of patient care. These are studies that involve algorithms whose outputs are directly used by clinicians in an organic or behavioral/mental health care setting (see the inclusion and exclusion criteria in Fig. 1). The PRISMA flowchart would naturally follow this explanation, as shown in Fig. 2, illustrating the flow of studies from identification to inclusion. Search strategy, study extraction and model analysis: a comprehensive literature search was conducted using the following databases: Ovid Embase, Ovid MEDLINE, Ovid PsycINFO, Web of Science Core Collection, Scopus and the Cochrane Library [8]. Study selection and data extraction: two reviewers used the Covidence software to screen all included abstracts’ titles, abstracts and full texts [9]. Two independent reviewers resolved disparities. Data extraction and quality assurance were conducted by all team members simultaneously using the Covidence software. At least two reviewers must agree with the data extracted for model inclusion. A third reviewer resolved conflicts to serve as a tiebreaker. Study endpoints: our endpoints were the percentages of published clinical AI/ML studies that reported the gender and racial composition of their training data. We were also interested in finding the number of studies that were conducted using open data registries. Open data are non-proprietary data that is accessible by everyone without the need for special permission.

## RESULTS

**Figure 3:**
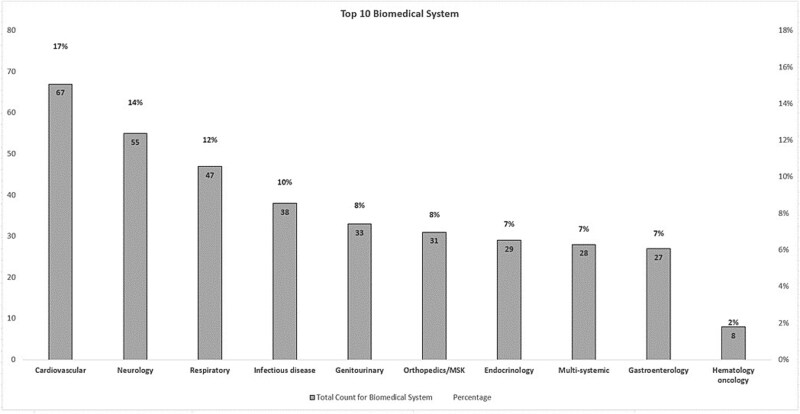
Biomedical system distribution of published clinical models.

**Figure 4:**
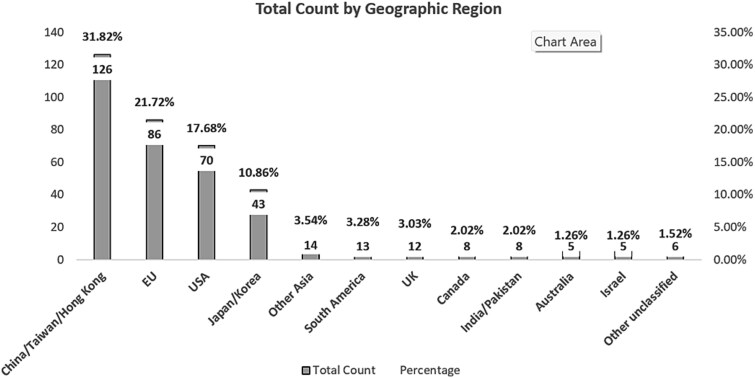
Geographical distribution of published clinical models.

Characteristics of the 390 included studies: the most prevalent biomedical systems in our review were Cardiology (67, 16.9%), Neurology (55, 13.9%) and Respiratory (47, 11.9%) (Fig. 3). Geographically, models in our sample were from China/Taiwan/Hong Kong (126, 31.82%), European Union (EU) (86, 21.72%), USA (70, 17.68%), Japan/Korea (43, 10.86%), Other Asia (14, 3.54%), South American (13, 3.28%), UK (12, 3.03%), Canada (8, 2.02%), India/Pakistan (8, 2.02%), Australia (5, 1.26%), Israel (5, 1.26%), Other unclassified (6, 1.52%) (Fig. 4) Only 16% of the models were built using accessible open data registries. Study endpoints: of the reviewed 390 AI/ML studies, 84% of global AI studies did not provide demographic composition based on ethnicity. In contrast, 44% of US-based studies did not provide ethnicity information in their training data. Thirty-one percent of published global models did not provide gender data, while 23% of US studies did not provide gender information in their training data, see Table 1.

**Table 1:** Primary endpoints: disclosure of gender and racial composition in AI/ML studies

Characteristics		Results, numbers (%)	
Break down by ethnicity		Worldwide	USA only
	Yes	61 (15.40)	39 (55.71)
	No	335 (84.60)	31 (44.29)
Break down by gender			
	Yes	272 (68.69)	54 (77.14)
	No	124 (31.31)	16 (22.86)

## DISCUSSION

Clinical AI/ML model applications are likely to continue increasing over the years, given the ever-growing trove of patients’ clinical and lifestyle data that can be easily accessed through electronic health records and open data sources, such as social media data. Our study emphasizes the need to ensure that global models and algorithms developed in the USA are built with representative data. However, models developed in the USA did relatively better, providing demographic data compared to global models. This is still rather disappointing, with almost half of the US models in our study (44%) lacking the ethnicity composition of their training dataset. Based on the increasing ethnic and racial diversity of the USA [10], it becomes a matter of patient safety to ensure training data sets are adequately representative. The implication of non-representative training data for building clinical models can result in direct patient harm and worsening health disparities and discrimination [11].

The advent of the large language models (LLMs) proliferation and deployment in healthcare settings following the launch of ChatGPT also calls for higher vigilance for validity and data integrity. LLMs and artificial neural networks are encoded and trained on gigantic data sets rife with societal prejudices and built-in inaccuracies. This played out in LLM’s differential diagnosis and treatment recommendations that are filled with inaccurate stereotypes of particular demographic groups, deepening the existing inequities [12]. As a society, the same mistakes made in past drug and device development appear to be recurring. Notably, the NIH did not mandate the inclusion of women and minorities in NIH-sponsored clinical trials until 1994 [13].

Our study also underscores the issue of a lack of transparency in AI/ML models [14]. This results in an inability to reproduce contemporary AI/ML studies even at the research stage of model development. This undermines the whole essence of publishing a model in an academic forum for verification. Only 16% of models in the sampled studies were built with open, non-proprietary data. This issue of proprietary data used to build models at the research stage is a precursor to upstream models that are eventually deployed. This creates a situation whereby there is no possibility of independently confirming their validity [14].

An important limitation of our study, worthy of mention here, is that our sample underrepresented studies from Africa and the Middle East, with an overrepresentation of studies from China/Taiwan/Hong Kong (31.82%). This could have skewed our findings in ways beyond our control.

## CONCLUSION

The absence of demographic information in a substantial number of studies raises concerns about these models’ generalizability and equitable application. However, there was a higher disclosure of gender and racial composition models published in the USA. Nevertheless, reporting gender and ethnicity composition in US models is still sub-par, considering the diverse nature of the US population compared to other geographical clusters worldwide. The high occurrence of models built on proprietary data, which limits independent verification and validation of model output, is also a significant concern for bias and patient safety. Addressing these issues is crucial for advancing the reliability and inclusivity of clinical AI and ML applications. We believe that including

women’s and minority data, as mandated by the NIH for drug and device development, will go a long way in alleviating this problem during the early phase of clinical AI model application. We recommend that funders and policymakers mandate standardized demographic reporting with minimum thresholds for representativeness as a prerequisite for funding AI studies. This is as recommended by reputable consensus guidelines from the World Health Organization (WHO), NIH and FDA [15–21].

## Supplementary Material

092623_HS-Latest_Model_Update_oqaf027

## Data Availability

The data supporting the findings of this study are available within the article and its supplementary materials. Where applicable, datasets generated or analyzed during this study are accessible on the supplemental (an Excel file was uploaded during the application process). For further details or inquiries about data access, please contact the corresponding author, Dr Ahmed umar Otokiti, at ahmedotoks@yahoo.com.
